# Petrology and geochemistry dataset of the volcanic scoriae from southern part of the continental Cameroon Volcanic Line

**DOI:** 10.1016/j.dib.2022.108624

**Published:** 2022-09-20

**Authors:** Paul-Desire Ndjigui, Estelle Huguette O. Ngono

**Affiliations:** Department of Earth Sciences, University of Yaoundé I, P.O. Box 812, Yaoundé, Cameroon

**Keywords:** Scoriae, Cameroon volcanic line, Petrological characterization, Valorization, Cement manufacture

## Abstract

Petrological and geochemical data of scoriae from the southern part of the continental Cameroon Volcanic Line were obtained through field and laboratory investigations. Field observations were done at nine localities (Limbe, Djoungo, Loum-Tombel, Manjo-Monengolle, Melong, Doupe, Yamba, Foumbot, Njinkouo, and Galim) under forest and savanna cover during the dry season in order to facilitate the collect of samples. This was based on some criteria such as the size (centimeter to metric) and the color (gray, brown, red, or black) of samples. During the same field works, samples were also collected. Microscopic observations were done using a Leica DM 750P optic microscope. Mineralogical assemblages of crystalline phases without the amorphous phases were obtained by X-ray diffraction (XRD) instrument. Geochemical data of major elements were acquired using the X-ray fluorescence spectrometry (XRF). The contents of some major elements and the evaluation of the chemical weathering intensity of scoriae are presented together in the ternary diagrams. The evaluation of intensity of chemical weathering was done using bivariate diagram CIW (Chemical Index of Weathering) versus CIA (Chemical Index of Alteration). Chemical bonds were observed using infrared spectra. These data provide chemical conditions of volcanic scoriae that must have to be used in civil applications such cement manufacture. Data of this paper are further presented and discussed in Ngono Onana et al. [1].

**Specifications Table**

**Table:** 

Subject	Earth Sciences
Specific subject area	Petrology, mineralogy and geochemistry
Type of data	Table, figures, graph and pictures
How the data were acquired	Field investigations: GPS map (Garmin 62S); sampling; Microscopic mineralogy: a Leica DM 750P microscope (University of Yaoundé 1); Mineral phases: X-ray powder diffraction (Geoscience Laboratories (Sudbury, Canada)); Major elements: X-ray Fluorescence (Geological Institute (ISTE) of the University of Lausanne (Switzerland) and Amorphous phases: Infrared (IR) spectrometry (University of Yaoundé I); binary and ternary diagrams.
Data format	Raw and analysed.
Description of data collection	The choice was made on samples of centimeter to metric size. Sampling was carried out based on the color (or facies) of the fresh rocks were selected for rock thin section-preparation and representative samples for whole rock analysis. The removal of the scoriae was done using a peel, a hammer and pickaxe. Larger samples were also collected. The samples were packed. Each sample was identified by a code indicating the locality of sampling and its color.
Data source location	The Southern part of the continental Cameroon Volcanic Line (CVL) as reported in [1]. These data were collected in four regions of Cameroon (Central Africa): Littoral, South West, West, and North West). Data concerning source location (latitude and longitude using GPS) were also presented in Table 1.
Data accessibility	Data are available in: Mendeley; Cameroon Volcanic Scoriae data; DOI: 10.17632/5jcfdc2yhs.1;URL: https://data.mendeley.com/datasets/5jcfdc2yhs/1
Related research article	Estelle Huguette Ngono Onana, Fuh Calistus Gentry, Paul-Desire Ndjigui. Petrological features of volcanic scoriae from the southern part of the Cameroon Volcanic Line and their supplementary cementations application. *Heliyon* 8, e08684 [1].

## Value of the Data

• Chemical data contribute to highlight an exploitation of these materials in the cement industry as raw materials.
• Data help to define some criteria of scoriae from Cameroon Volcanic Line as raw materials in the cement manufacture.
• Dataset contributes to establish a link between the chemical composition and the technical properties of scoriae in the cement industry.
• Data provide additional knowledge on the petrological features of the Cameroon Volcanic Line products.
• Mineralogy data highlight the reactive phase and the mineral assemblage of the crystalline phase.
• Data enable to recognition of suitable and no suitable scoriae in the cement manufacture.

## 1 Data Description

**Fig. 1:**
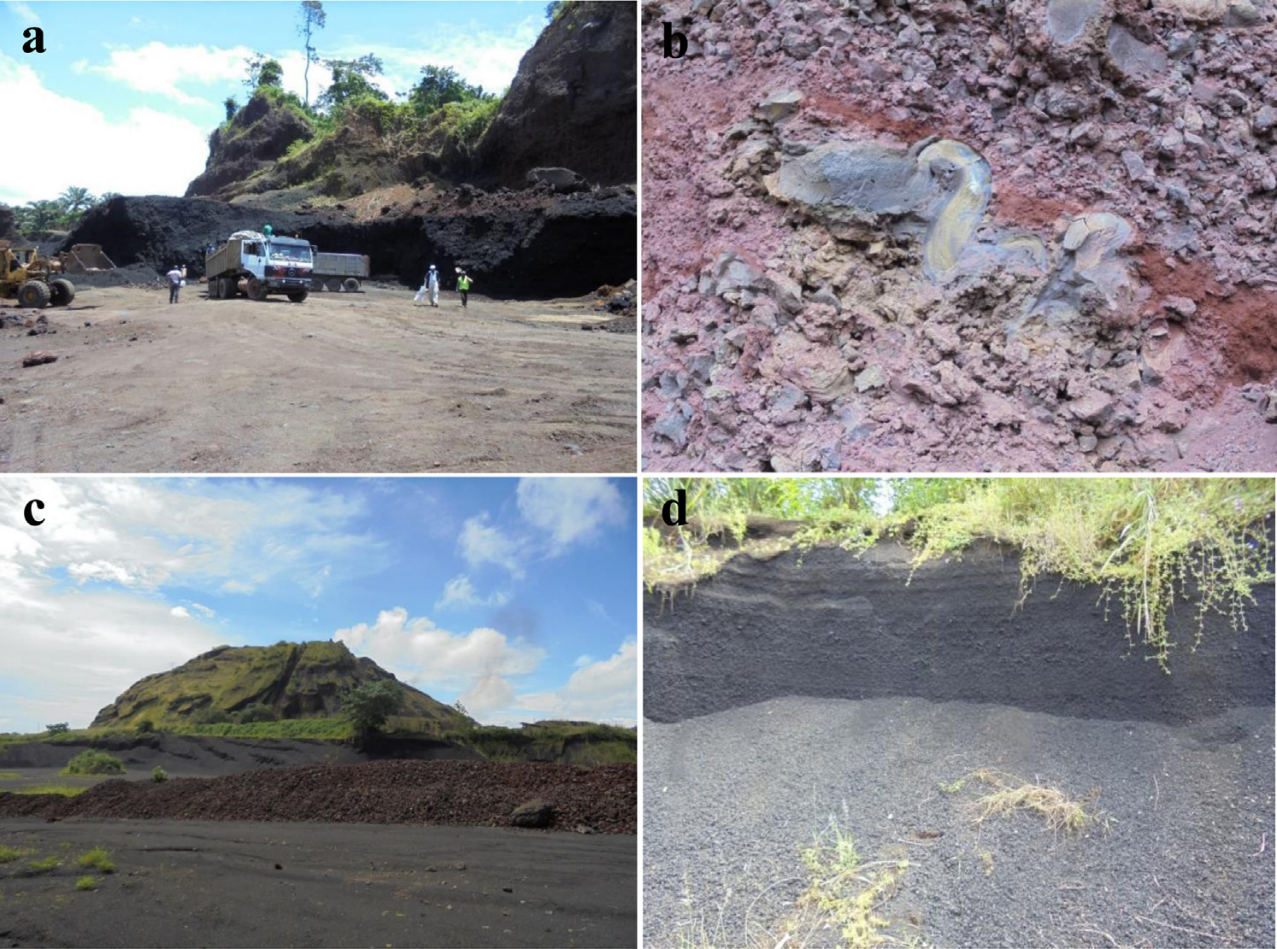
Macroscopic observations of scoriae from the Cameroon Volcanic Line: a) Limbe quarry with black type, the presence of transport and collect appliances for volcanic scoriae indicates their valorization in the civil application works; b) Red scoriae with different sizes in the Limbe quarry; 1) Djoungo quarry with the red facies; d) Galim black scoriae with loose texture.

**Fig. 2:**
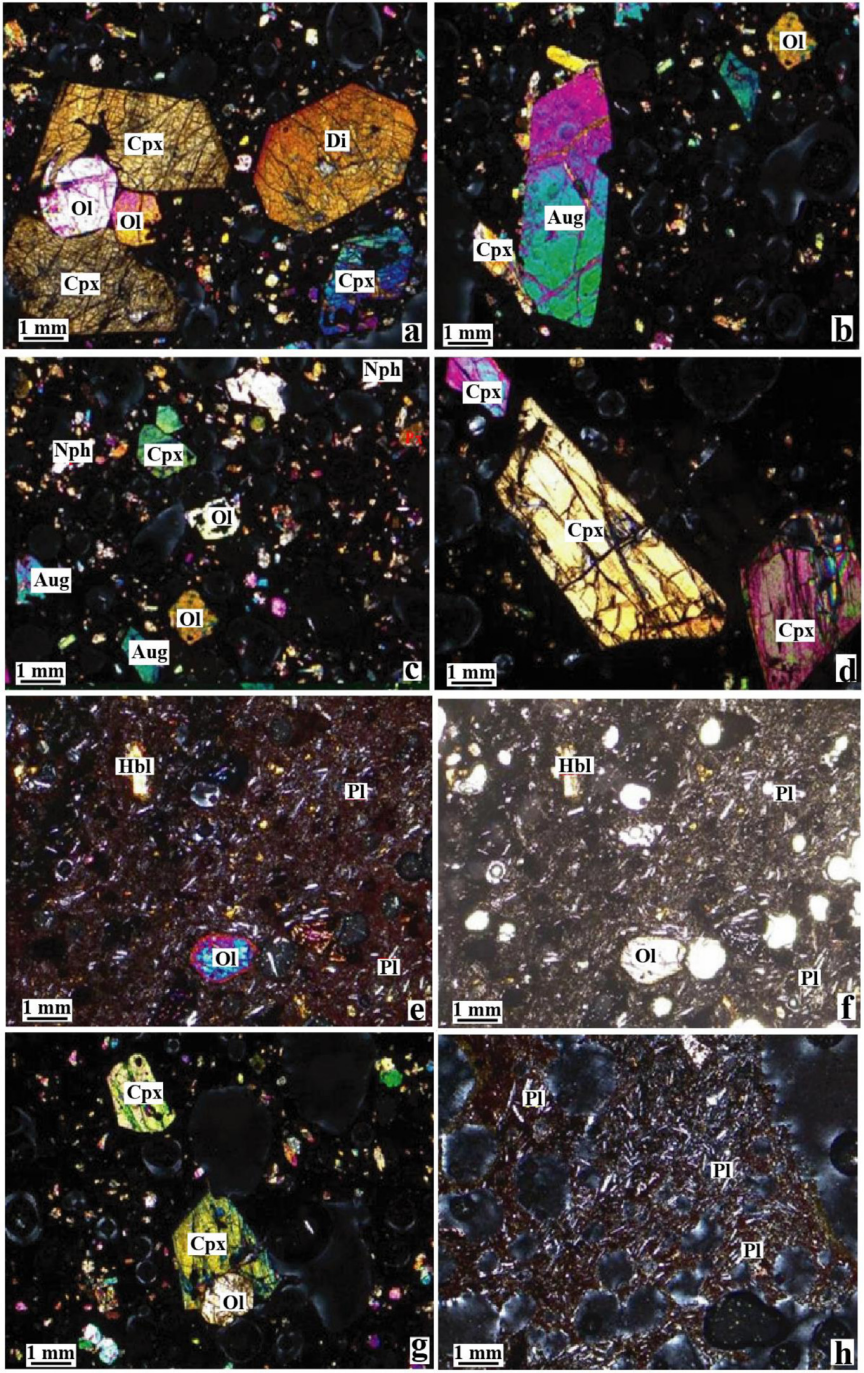
Photomicrographs showing some mineral assemblages of basanite (a – d) and basalt (e – h): Ol = olivine; Cpx = clinopyroxene; Di = diopside; Aug = augite; Nph = Nepheline; Hbl = hornblende; Pl = plagioclase. Figs. 2(a –c) showing the phenocrysts of clinopyroxene and olivine; Fig. 2d showing the amphibole growth after clinopyroxene; Fig. 2e presents the aureole of the weathering of olivine into iddingsite (a-e) and (g-h) in XPL; f in PL.

**Fig. 3:**
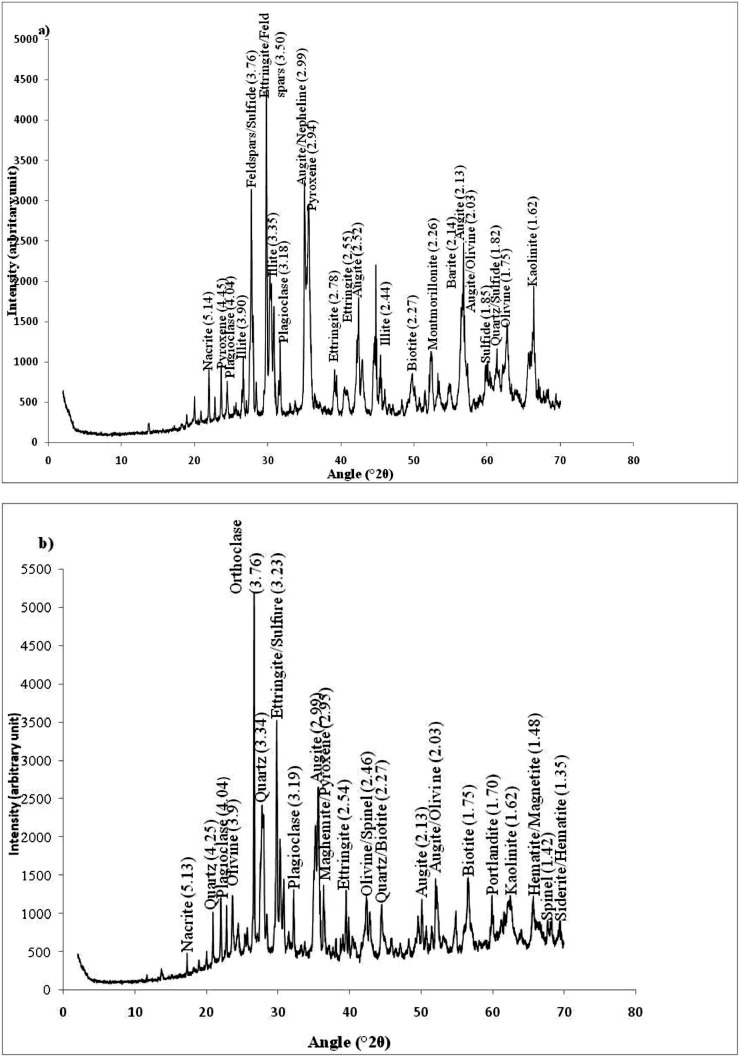
X-ray Diffraction (XRD) spectra of crystalline phases without the amorphous phases.

**Fig. 4:**
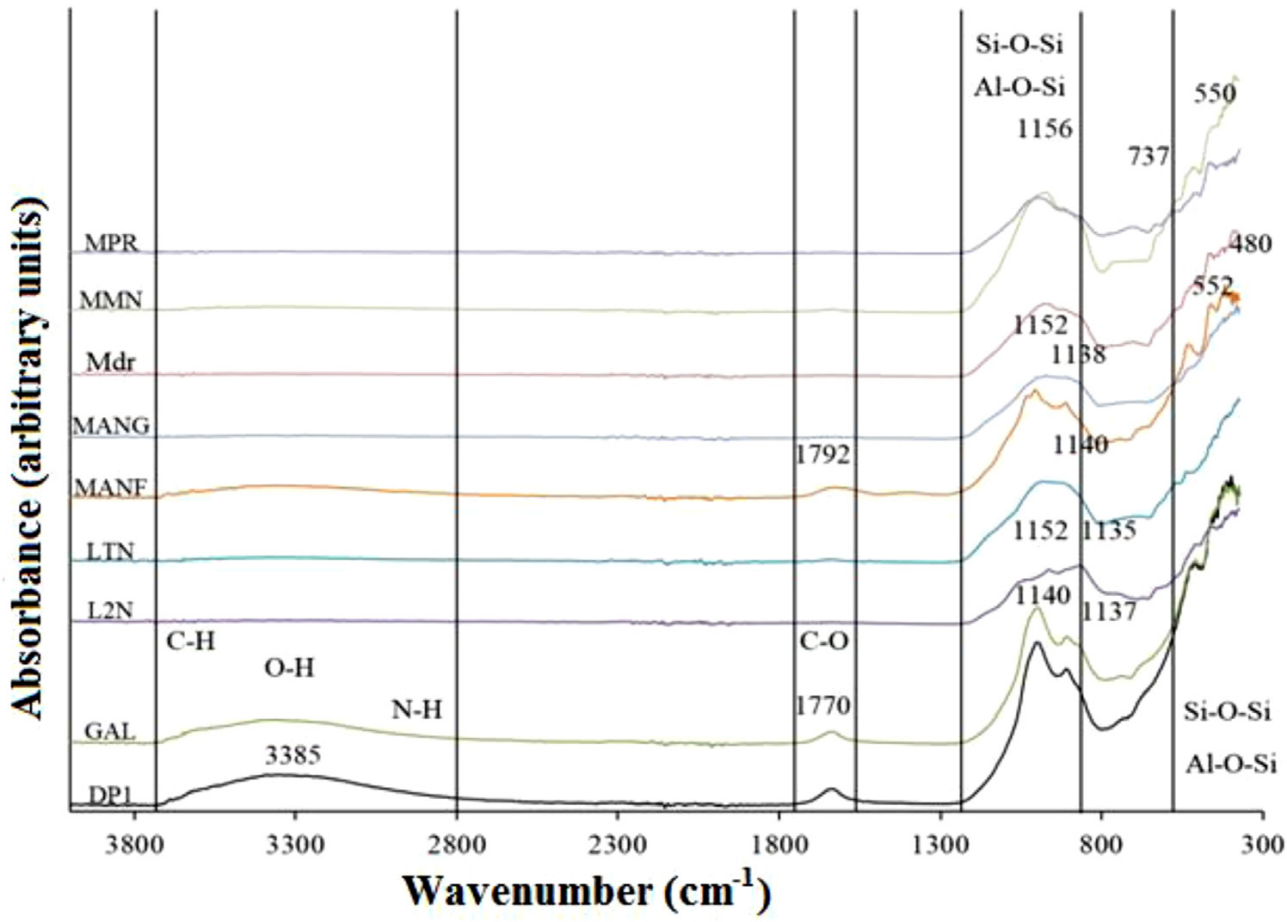
Fourier transform infrared (FT-IR) spectra showing various bonds of crystalline phases.

**Fig. 5:**
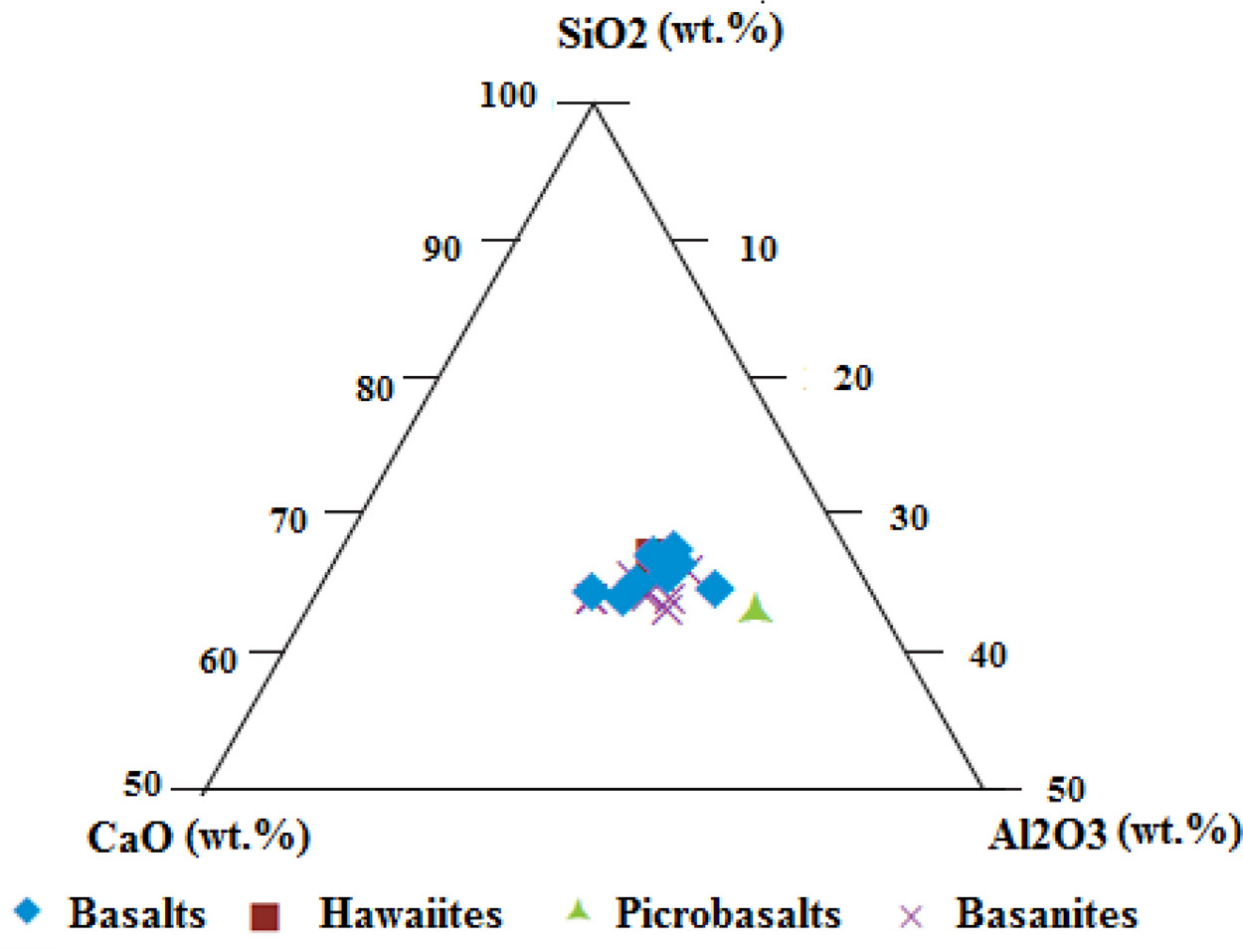
Plotting of scoriae from the Cameroon Volcanic Line in the CaO-SiO_2_-Al_2_O_3_ diagram.

**Fig. 6:**
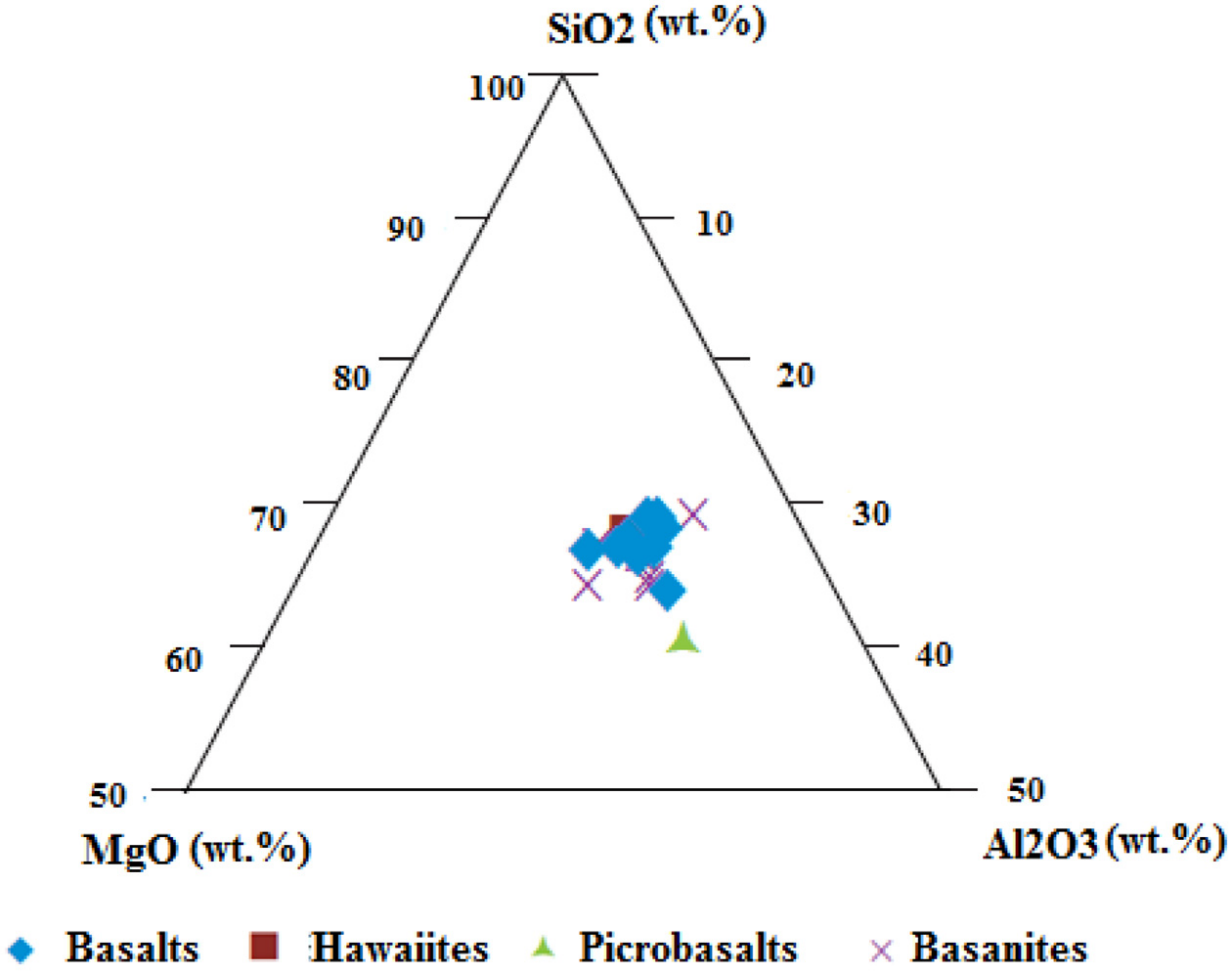
Plotting of scoriae from the Cameroon Volcanic Line in the MgO-SiO_2_-Al_2_O_3_ diagram.

**Fig. 7:**
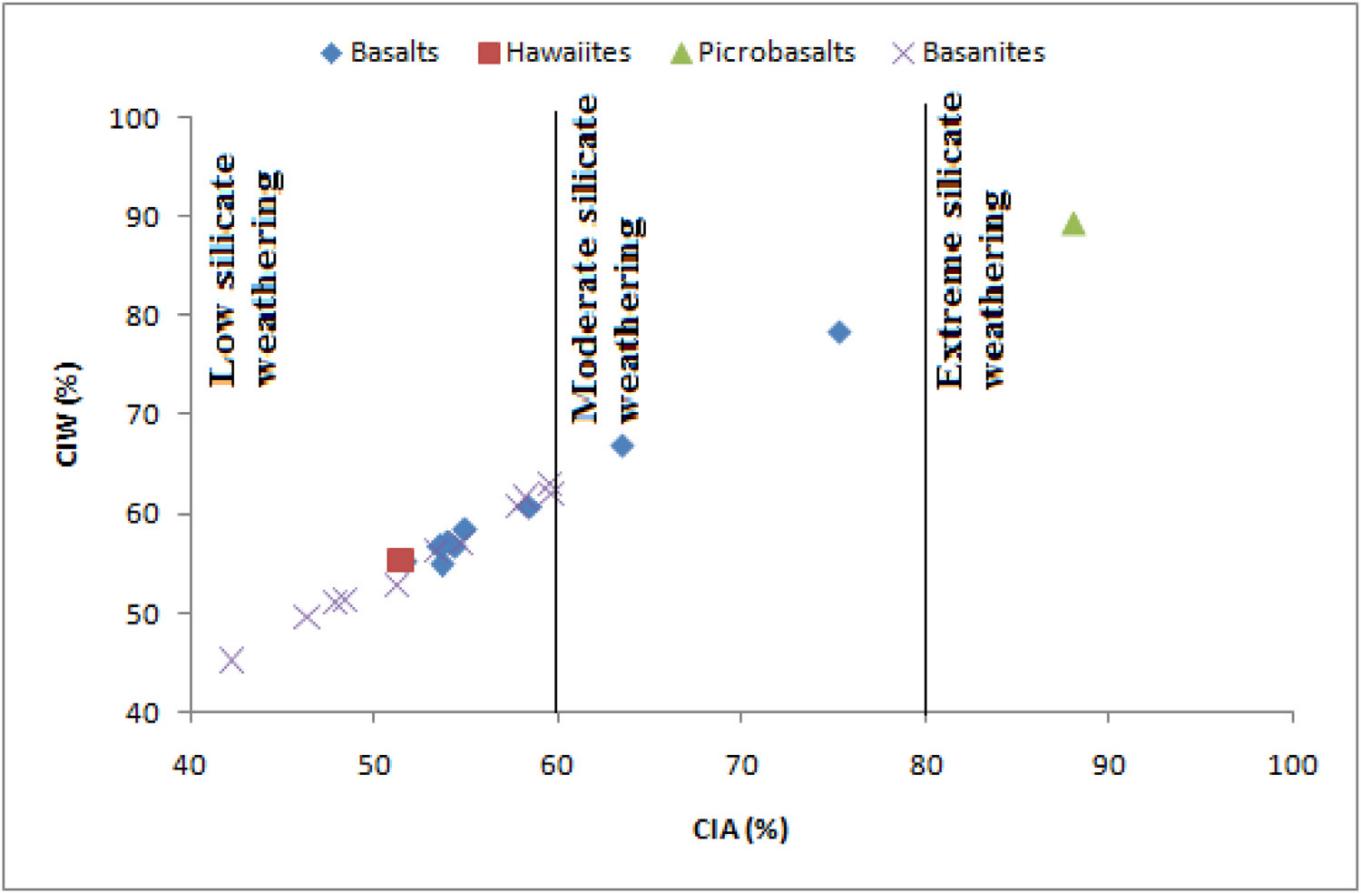
Variation of the intensity of weathering in the Chemical Index of Alteration (CIA) versus Chemical Index of Weathering (CIW) diagram.

The data presented are those of volcanic materials collected in tropical environment (West Cameroon) during the dry season. This paper provides information on the volcanic scoriae in the field and mineral assemblages (Fig. 1, Fig. 2), crystalline and amorphous phases (Fig. 3, Fig. 4 and supplementary material files: Tables i and ii), and their geochemical nature (Fig. 5, Fig. 6, Fig. 7 and supplementary material files: Tables iii and iv). Furthermore this article displays that the volcanic scoriae can be used during civil application works (see Fig. 1a) and also as cement replacement owing to their chemical compositions (Fig. 3, Fig. 4, Fig. 5, Fig. 6, Fig. 7). The sampling points referenced from geographic coordinates are reported in Table 1.

**Table 1:** Geographical coordinates of volcanic scoriae from the southern part of the Cameroon Volcanic Line.

Sites	Sample code	Geographical coordinates
LIMBE	L1R	N04°1′99.3′’
	L1N	E09°10′44.3′’
	L2R	
	L2N	N04°1′39.5′’
	L2G	E09°10′44.4′’

DJOUNGO	MDR	N04°35′03.2′’ E09°37′44.5′’
	MDN	N04°34′57.4′’ E09°37′30.5′’

LOUM-TOMBEL	LTN	N04°43′21.5′’ E09°41′26.0′’
	LTR	

MANJO-MONENGOLLE	MANJ	N04°51′42.1′’ E09°50′18.7′’
	MANR	

MELONG	MMR	N05°03′11.8′’ E09°53′44.3′’
	MMN	
	MPR	N05°01′54.8′’ E09°55′33.5′’

DOUPE	DP1	N05°30′54’’ E10°31′53’’
	DP2	N05°30′58’’ E10°31′46’’

YAMBA	YMB1	N05°28′56’’ E10°29′55’’
	YMB2	N05°28′52’’ E10°31′05’’

FOUMBOT	MANG	N05°28′13’’ E10°37′44’’
	MANF	N05°29′05’’ E10°37′26’’

NJINKOUO	NJK1	N05°28′42’’ E10°37′03’’
	NJK2	

GALIM	GAL	N05°39′01’’ E10°24′25’’

## 2 Experimental Design, Materials and Methods

Scoria was sampled in accessible quarries and outcrops along the southern continental part of Cameroon Volcanic Line. Several field works were conducted to collect samples during the dry season (March 2016) in order to facilitate the accessibility to different study sites. During the field works, samples were described, packed and identified by a code indicating the sampling point and the color of each sample. This sampling protocol is very simple and similar for all mining investigations on loose geological materials. Samples were collected from the southern part of the continental Cameroon Volcanic Line, in nine localities (Table 1).

The selected samples were used to the manufacture of rock thin sections. The manufacture of these thin sections comprises six steps after the consolidation using the Canada balsam glue: sawing or slab cutting, initial lapping of the slab, addition of glass slide, sawing of the prepared slab, final lapping, cleaning of the thin sections. The detail descriptions are available on https://elearn.univ-tlemcen.dz or https://LakeheadUniversity.com. Observations of the rock thin sections under the microscope allow documenting mineral occurrence and textural features such as mineral reactions (amphibole after clinopyroxene). The volcanic scoriae have basanitic, hawaiitic and basaltic compositions, exhibit glassy texture and are essentially composed of plagioclase, olivine, clinopyroxene, ± hornblende, while nepheline is noticed as secondary mineral.

Samples were air-dried crushed and finely ground with a grinder to a required fine powdered sample (2 mm sieve). For mineralogical, geochemical and Fourier transform infrared IR spectrometry (FT-IR) analyses, samples were sieved at 250 µm.

Mineralogical and geochemical analyses (trace elements) were carried out at the Geoscience Laboratories (Sudbury, Canada). The mineralogical composition was determined using the PAN Analytical X'PERT PRO diffractometer equipped with a monochromator. Sample powders were pulverized with an agate mortar and pestle (2 or 3 g) and smear mounts were prepared on low background silicon disks for analysis. Kα_2_ peaks shown as dashed lines and do not have a ‘’V-shaped’’ indicator at the top of the pattern windows. Kα_2_ peaks have been omitted from the peak list to facilitate the interpretation of the samples. Samples were analysed with Co radiation at 40 kV and 45 mA. The following parameters were used in the X'Pert High Score Plus software for the peak identification: Minimum significance: 1.00; Minimum tip width (°2θ):0.01; Maximum tip width (°2θ): 1.00; Peak base with (°2θ): 2.00; Method: Top of smoothed peak. These data were reported under the certificate number CRT-18–0421–01 of 28.01.2019. All raw data and QC certificate are available as supplementary material files (Tables v and vi). This spectrometer was used to determine a set crystalline phases (feldspar/sulfide, quartz, augite/olivine, ettringite/feldspars, augite/nepheline, olivine, kaolinite, sulfide, illite, plagioclase, augite and pyroxene) on samples.

The determination of major elements concentrations was done at the Geological Institute (ISTE) of the University of Lausanne (Switzerland). Powders (2 or 3 g) were analyzed by X-Ray Fluorescence Spectrometry using a PANalytical PW2400 wavelength dispersive spectrometer to obtain the composition of SiO_2_, TiO_2_, Al_2_O_3_, CaO, Fe_2_O_3_, MgO, MnO, Na_2_O, K_2_O, Cr_2_O_3_, NiO and P_2_O_5_. A two-step loss on ignition (LOI) determination was employed: 105 °C under nitrogen to drive off adsorbed water; 1000 °C under oxygen to drive remaining volatiles and oxidize Fe. The LOI measurement was done before the determination of major element concentrations using XRF instrument. Two international standards (Sy-2 and NIM-N) were used (supplementary material file: Table vii).

The determination of trace elements (including rare earth elements) was done using an Inductively Coupled Plasma-Mass Spectrometry (ICP-MS) instrument. Powders (0.25 g) were digested with a mixture of two acids (HCl + HClO_4_) at 120 °C in sealed Teflon containers during one week, after, rinsed with dilute HNO_3_ and dried. The residue was again dissolved in a mixture of three acids (HNO_3_, HCl, and HF) at 100 °C. Two types of reference materials were used: the InHouse Reference Material (ISHT-18–25,786, 18–25,787, and 18–25,788) with the QC Name MRB-29 and the International Reference Material (INTL-18–31,560 (QC Name: GSP-2), 18–31,561, and 18–31,562) with the QC Name GSP-2. The Certificate Ontario Reference Material (CORM) was also used. The Quality Control (QC) used also the Instrument Control (INST), the Laboratory Duplicate (DUP-18–45,795, 18–45,795, 18–45,796, and 18–45,797; the QC Name is DUP) and the Laboratory Blank (BLANK-18–19,493, 18–19,494, and 18–19,495). The raw data of QC are reported in supplementary material file (Table viii).

The Fourier transform infrared IR spectrometry (FT-IR) analyses were used for the measurement of the vibrations of atoms and the determination of the functional groups. The Fourier transform infrared (FT-IR) spectrometry data were obtained at the Inorganic Laboratory of the University of Yaoundé I (Cameroon) using the BURKER OPTIK ALPHA spectrometer. The well crystallized kaolinite (KGa-1b) and the Polystyrene infrared standard were used as International Standards. Well crystallized kaolinite was obtained from the Source Clay Repertory of the Clay Minerals Society (Purdue University, West Lafayette, Indiana, USA). Polystyrene infrared standard helps to compare the spectra with those of the data base. For this analysis, we used 0.2 g of sample just to cover the diamond. This spectrometer was used to obtain various spectra of crystalline phases and theirs behavior with C-H, O-H, N-H, C-O, Si-O-Si and Al-O-Al functions. The infra-red spectra were recorded in the wavelength range from 4000 to 400 cm^−1^. Absorbance and wave number plot of these volcanic scoriae exhibits flat spectra without any wave concerning mono-boundary crystalline phases whereas into di-boundary crystalline phases (Si-O-Si and Al-O-Al) with low wave numbers, many fluctuations occur suggesting that the amorphous phase in the sample contained calcium ions that hinder the consumption of calcium from ettringite, feldspars and plagioclase which are present in the volcanic scoriae.

Basanites and hawaiite are low silicate weathering, basalts are low to moderate silicate weathering while picrobasalt is extreme silicate weathering. However, The Chemical Index of Alteration (CIA) and Chemical Index of Weathering (CIW) values highlight the low intensity of chemical weathering [2,3]. Thus, these volcanic scoriae are amorphous phase and k-feldspar high content suggesting their possible use as cement replacement during civil applications. This trend is confirmed by SiO_2_-CaO-Al_2_O_3_ and SiO_2_-MgO-Al_2_O_3_ diagrams where SiO_2_, CaO, MgO and Al_2_O_3_ vary between 60–70%, 30–40%, 30–40% and 30–40%, respectively. All these indicate that volcanic scoriae from the southern continental part of the Cameroon volcanic line can serve as cement replacement during civil applications.

## 3 Geology

The volcanic scoriae studied are located at the southern part of Cameroon Volcanic Line which represents SSW to NNE trending. This Cameroon volcanic line is made up of oceanic and continental volcanic mountains and orogenic plutonic complexes. The studied area belongs to the continental part. The Cameroon Volcanic Line might be the result of a SW–NE linear mantle upwelling, or ‘hotline’, possibly developed along lithospheric discontinuities during reactivation of Pan-African lineaments between the cratonic blocks [4].

## CRediT authorship contribution statement

**Paul-Desire Ndjigui:** Conceptualization, Project administration, Resources, Supervision, Data curation, Formal analysis, Visualization, Writing – review & editing, Validation. **Estelle Huguette O. Ngono:** Methodology, Data curation, Formal analysis, Investigation, Writing – original draft, Writing – review & editing.

## Declaration of Competing Interest

The authors have no conflicts of interests. The authors agree with the information provided in this manuscript and the Data in Brief policy.

## Data Availability

Petrology and geochemistry dataset of the volcanic scoriae from southern part of the continental Cameroon Volcanic Line (Original data) (Earth/Chem).
